# *In silico, in vitro* and cellular analysis with a kinome-wide inhibitor panel correlates cellular LRRK2 dephosphorylation to inhibitor activity on LRRK2

**DOI:** 10.3389/fnmol.2014.00051

**Published:** 2014-06-03

**Authors:** Renée Vancraenenbroeck, Joren De Raeymaecker, Evy Lobbestael, Fangye Gao, Marc De Maeyer, Arnout Voet, Veerle Baekelandt, Jean-Marc Taymans

**Affiliations:** ^1^Laboratory for Biomolecular Modelling, Division of Biochemistry, Molecular and Structural Biology, Department of Chemistry, KU LeuvenLeuven, Belgium; ^2^Laboratory for Neurobiology and Gene Therapy, Department of Neurosciences, KU LeuvenLeuven, Belgium; ^3^Zhang Initiative Research Unit, RikenSaitama, Japan

**Keywords:** docking, MOE, LRRKtide, Parkinson's disease, kinase, phosphorylation, inhibitor, receiver operator characteristic

## Abstract

Leucine-rich repeat kinase 2 (LRRK2) is a complex, multidomain protein which is considered a valuable target for potential disease-modifying therapeutic strategies for Parkinson's disease (PD). In mammalian cells and brain, LRRK2 is phosphorylated and treatment of cells with inhibitors of LRRK2 kinase activity can induce LRRK2 dephosphorylation at a cluster of serines including Ser910/935/955/973. It has been suggested that phosphorylation levels at these sites reflect LRRK2 kinase activity, however kinase-dead variants of LRRK2 or kinase activating variants do not display altered Ser935 phosphorylation levels compared to wild type. Furthermore, Ser910/935/955/973 are not autophosphorylation sites, therefore, it is unclear if inhibitor induced dephosphorylation depends on the activity of compounds on LRRK2 or on yet to be identified upstream kinases. Here we used a panel of 160 ATP competitive and cell permeable kinase inhibitors directed against all branches of the kinome and tested their activity on LRRK2 *in vitro* using a peptide-substrate-based kinase assay. In neuronal SH-SY5Y cells overexpressing LRRK2 we used compound-induced dephosphorylation of Ser935 as readout. *In silico* docking of selected compounds was performed using a modeled LRRK2 kinase structure. Receiver operating characteristic plots demonstrated that the obtained docking scores to the LRRK2 ATP binding site correlated with *in vitro* and cellular compound activity. We also found that *in vitro* potency showed a high degree of correlation to cellular compound induced LRRK2 dephosphorylation activity across multiple compound classes. Therefore, acute LRRK2 dephosphorylation at Ser935 in inhibitor treated cells involves a strong component of inhibitor activity on LRRK2 itself, without excluding a role for upstream kinases. Understanding the regulation of LRRK2 phosphorylation by kinase inhibitors aids our understanding of LRRK2 signaling and may lead to development of new classes of LRRK2 kinase inhibitors.

## Introduction

Leucine-rich repeat kinase 2 (LRRK2) is a 2527 amino-acid long complex multidomain protein which is a member of the ROCO protein family. This family of proteins is derived from a signature homologous region including a domain encoding for a GTPase of the Ras family, termed ROC (for Ras of complex proteins) (Taymans, 2012), followed by a characteristic COR (C-terminal of ROC) domain. The ROC-COR bidomain is flanked C-terminally by a kinase domain and a WD40 domain and N-terminally by an armadillo repeat domain (ARM), ankyrin repeat domain (ANK), and the namesake leucine-rich repeat (LRR) domain. LRRK2 has primarily been studied for its role in Parkinson's disease (PD) (Cookson, 2010), but is also reported to play a role in cancer, Crohn's disease, and leprosy (Lewis and Manzoni, 2012). LRRK2 is the single most prevalent genetic cause of PD known to date (Paisan-Ruiz et al., 2008). Together with alpha-synuclein, LRRK2 has been both linked to familial PD and associated to sporadic PD (Singleton et al., 2013). Also, PD patients carrying the LRRK2 mutations show a clinical and neuropathological profile which is indistinguishable from sporadic PD, indicating that LRRK2 may contribute to a PD pathway common to both familial and sporadic PD (Healy et al., 2008).

The kinase activity of LRRK2 has been proposed as a promising target for developing disease modifying therapy for PD (Greggio and Singleton, 2007; Vancraenenbroeck et al., 2011; Lee et al., 2012) and deletion of LRRK2 kinase activity has been shown to be protective in cellular (Greggio et al., 2006; Smith et al., 2006) or *in vivo* models (Lee et al., 2010; Yao et al., 2013) of LRRK2 mediated toxicity. Currently, several compounds have been reported that are capable of inhibiting LRRK2 kinase activity (reviewed previously; Vancraenenbroeck et al., 2011; Deng et al., 2012; Kramer et al., 2012). Of these examples, staurosporine, K252A, and sunitinib are promiscuous kinase inhibitors, known to bind several classes of kinases. Other described compounds are active on specific classes of kinases such as Ro-31-8220, H1152, and Y-27632 (Davies et al., 2000; Bain et al., 2007). Recently, several inhibitors for LRRK2 with an *in vitro* potency in the low nanomolar range have been described including LRRK2-IN1 (Deng et al., 2011), CZC-25146 (Ramsden et al., 2011), TAE684 (Zhang et al., 2012), GSK2578215A (Reith et al., 2012), or HG-10-102-01 (Choi et al., 2012). These compounds are currently being implemented as tool compounds in basic research studies on LRRK2 and indicate the feasibility of developing LRRK2 inhibitors for other applications such as implementation as an imaging tracer or clinical testing.

One key question in assessing LRRK2 kinase inhibitors for these various applications involves understanding the molecular consequences of kinase inhibition in cells. Some clues are given recently from the effects of various inhibitors on the phosphorylation state of LRRK2 in cells. LRRK2 is a highly phosphorylated protein in cells with a notable cluster of phosphorylation sites in the interdomain region between the ANK and LRR domains, including sites Ser910/S935/S955/S973 (West et al., 2007; Gloeckner et al., 2010; Nichols et al., 2010; Lobbestael et al., 2012). Interestingly, these sites are dephosphorylated in cells or tissues following treatment by inhibitors of LRRK2 kinase activity (Dzamko et al., 2010; Choi et al., 2012; Doggett et al., 2012). It is tempting to conclude from these studies that phosphorylation levels at these sites reflects LRRK2 kinase activity, however kinase-dead variants of LRRK2 (K1906M or D2017A) or kinase activating variants (G2019S, T2031S) do not display altered Ser935 phosphorylation levels compared to wild type (Nichols et al., 2010; Lobbestael et al., 2013). Furthermore, Ser910/935/955/973 are not autophosphorylation sites but are rather sites phosphorylated by other kinases (West et al., 2007; Dzamko et al., 2010; Gloeckner et al., 2010; Nichols et al., 2010; Doggett et al., 2012), therefore, it is unclear if inhibitor induced dephosphorylation of LRRK2 wildtype depends on the activity of compounds on LRRK2 or on yet to be identified upstream kinases. We have recently shown that LRRK2 regulates its own dephosphorylation through protein phosphatase 1, including dephosphorylation induced by the LRRK2 kinase inhibitor LRRK2-IN1 (Lobbestael et al., 2013). It remains to be verified that LRRK2 inhibitor-induced dephosphorylation is generalized across multiple chemical classes and whether dephosphorylation is correlated to inhibitor binding to LRRK2 kinase.

In the present study, we addressed these issues using a chemical biology approach. A panel of cell permeable kinase inhibitors targeting all branches of the kinome was tested for its activity on LRRK2 *in vitro* as well as in cells. Using an optimized LRRK2 kinase homology model, selected compounds were docked *in silico* to assess binding at the ATP-binding site.

## Results

### Testing of a kinase inhibitor panel on LRRK2 *in vitro* kinase activity

**Figure 1:**
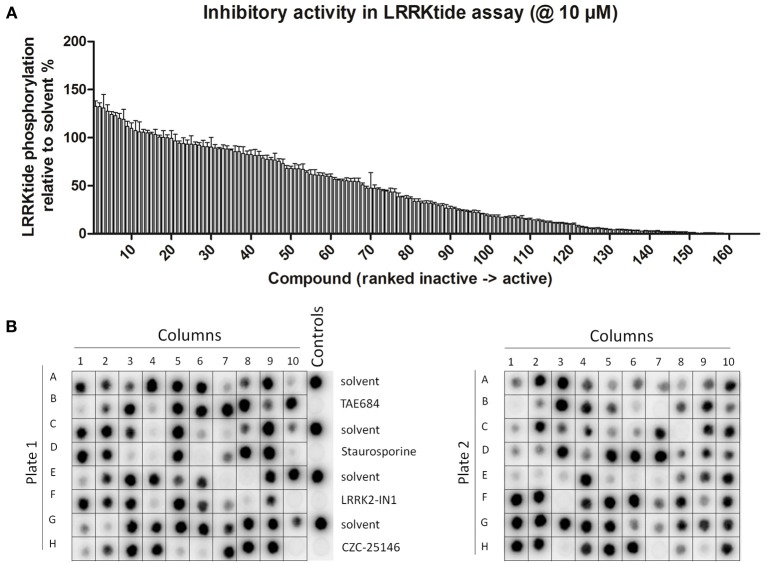
**Testing of effect of reference kinase inhibitors on *in vitro* LRRK2 kinase activity. (A,B)** 160 kinase inhibitors from a panel of inhibitors known to target kinases in all branches of the kinome were tested for their ability to inhibit LRRK2 at 10 μM in an *in vitro* kinase assay using the LRRKtide model peptide substrate, as described in Materials and Methods. **(A)** Quantification of the LRRKtide phosphorylation level for each kinase reaction. Signal intensity per reaction was quantified via densitometry as described in Materials and Methods and values are normalized to phosphorylation levels measured in solvent controls (control values are set at 100%). Values obtained (mean ± s.e.m., *N* = 3) are depicted as histogram bars ordered from least active to most active compound, showing that the panel comprises a broad range of activity on LRRK2 kinase function. Exact values are given in Table 1. **(B)** Representative autoradiograms of P81 paper spotted with kinase reactions from testing of the 160 compounds which were used for densitometric quantification given in A. Also shown at the right of the first panel are the solvent controls as well as positive controls using potent inhibitors of the LRRK2 kinase, TAE684, staurosporine, LRRK2-IN1, and CZC-25146. Illustration of detailed IC_50_ determination for active compounds is given in Figure 2.

**Figure 2:**
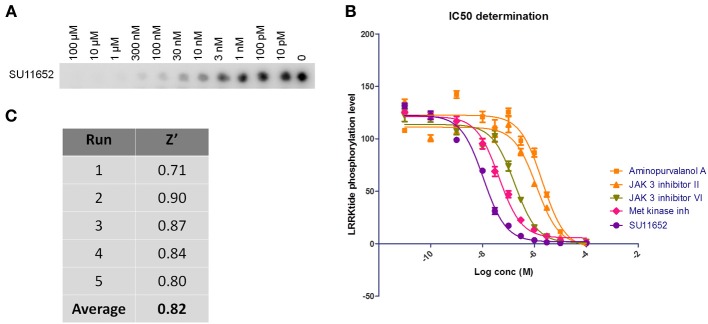
**Dose-response curves and Z' factor determination for *in vitro* LRRKtide assay. (A,B)** Detailed IC_50_ determinations were performed for selected active compounds by testing LRRKtide phosphorylation in the presence of a range of inhibitor doses, as described in Materials and Methods. A representative autoradiogram for compound SU11652 is given **(A)** as well as the fitted inhibition curves obtained for five compounds **(B)** *In vitro* IC50 values for each tested compound are given in Table 2. **(C)** LRRKtide phosphorylation values were used to calculate the Z' of the *in vitro* LRRKtide phosphorylation assay using the formula given in Materials and Methods.

The assay employed here is based on phosphorylation of a peptide termed LRRKtide derived from the cytoskeleton-associated moesin protein (Jaleel et al., 2007) and is adapted to a phosphor imaging readout (Asensio and Garcia, 2003; Taymans et al., 2011), as described in Materials and Methods and shown in Figure 1. The quality of the chosen assay is given by the average Z' factor for this assay which we determined to be 0.82 (Figure 2), well within the range of 0.5–1 which is considered an excellent value for screening assays (Zhang et al., 1999). A panel of 160 kinase inhibitors was tested in the LRRK2 *in vitro* kinase activity assay using GST-LRRK2r_970−2527_ at one concentration (10 μM) (Figure 1, quantifications given in Table 1). Of these 160 compounds, 35 compounds from three compound classes (A) 9-methyl-N-phenylpurine-2,8-diamine, (B) N-phenylquinazolin-4-amine, and (C) 1,3-dihydroindol-2-one analogs were selected for further testing in the *in vitro* assay as well as for *in silico* analysis (see further). These three compound classes were chosen because they contain compounds with a common core or scaffold possessing a wide variety of activities. Additionally, known LRRK2 reference compounds were tested including LRRK2-IN1, CZC-25146 and the pan-kinase inhibitor staurosporine as well as compound CDK1/2 inhibitor III which displayed highest potency in inhibiting LRRK2. The selected compounds were retested with full length recombinant enzyme at 100 μM, 10 μM, 1 μM and 100 nM. For those compounds that inhibited LRRK2 kinase activity more than 50% at 1 μM, a pIC_50_ [=−log(IC50)] value was determined as described in Materials and Methods (Figure 2 and Table 2). It can be noted that for known LRRK2 inhibitory compounds that we included in our testing, our adapted LRRKtide phosphorylation assay yielded similar potencies to those previously published (comparative examples of obtained IC50 values are: LRRK2-IN1 3.54 nM vs. 13 nM (Deng et al., 2011), CZC-25146 1.78 nM vs. 4.76 nM (Ramsden et al., 2011), with values given from this study and from published studies, respectively; for the LRRK2-IN1 comparison, the slightly lower value obtained here may be due to the ATP concentrations used which are 10 μM in the present study and 100 μM in the original study).

**Table 1:**
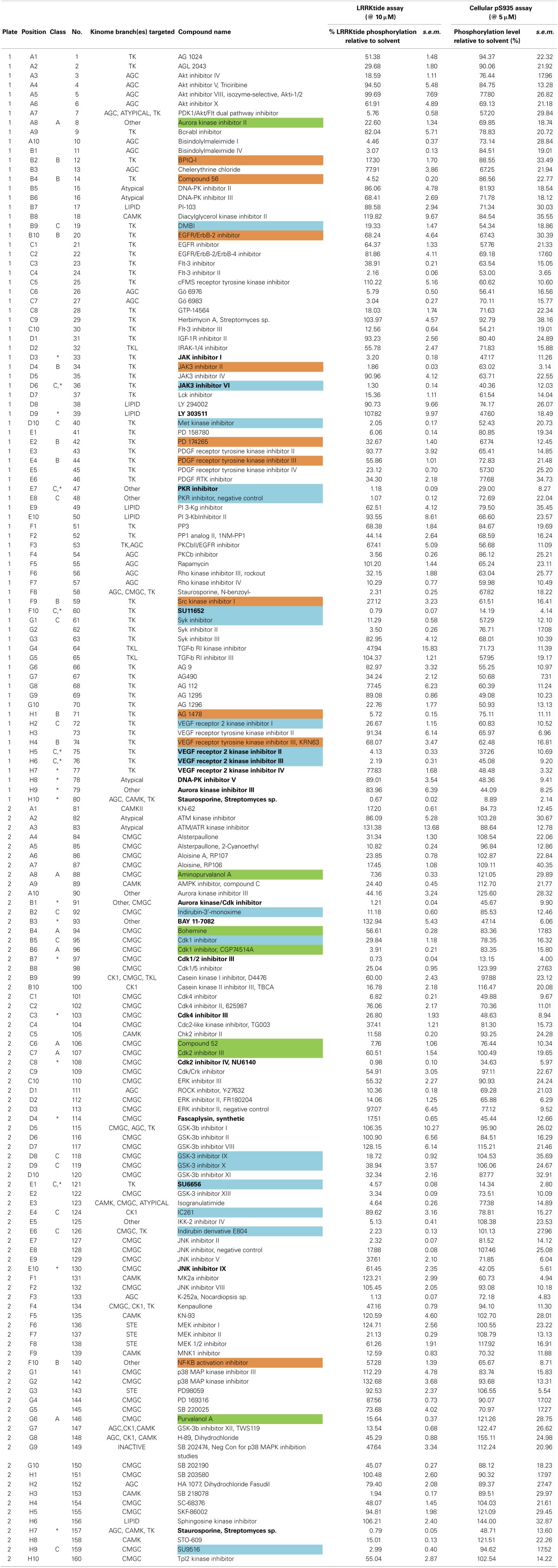
**Overview of *in vitro* and cellular activities of 160 kinase inhibitors tested at 10 and 5μM, respectively**.

**Figure 3:**
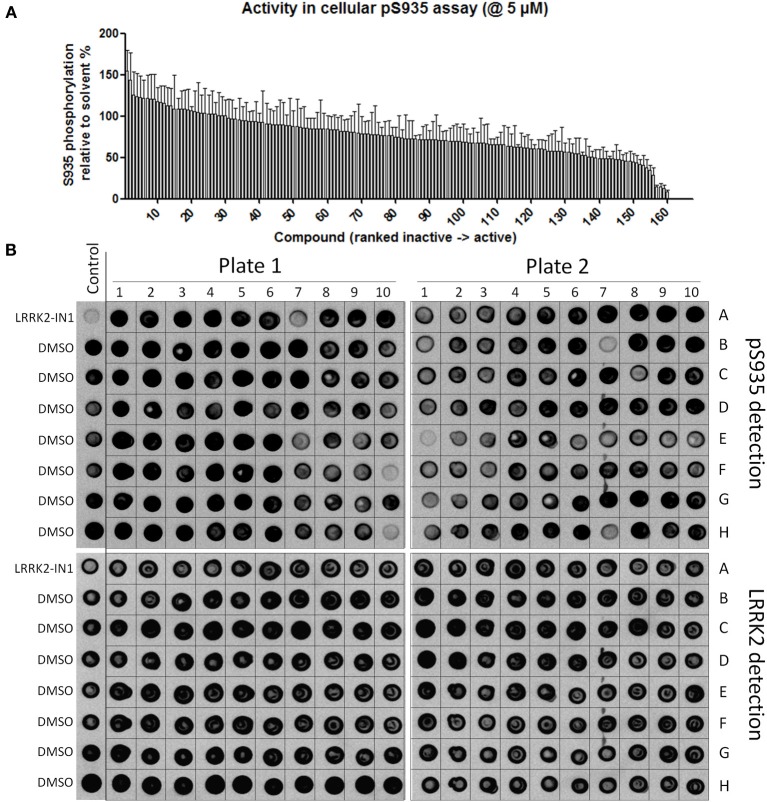
**Testing of effect of reference kinase inhibitors on cellular LRRK2 pS935 levels.** 160 kinase inhibitors from a panel of inhibitors known to target kinases in all branches of the kinome were tested for their ability to dephosphorylate LRRK2 at phosphoserine 935 in a spotblot assay, as described in Materials and Methods. **(A)** Quantification of the cellular phosphorylation level at Ser935 for each compound and ranked from least to most active (exact values per compound can be found in Table 1). **(B)** Representative spot blots detecting phospho-LRRK2 (pS935 detection in upper panels) and total-LRRK2 (LRRK2 detection in lower panels) for 160 kinase inhibitors tested at 5 μM. Illustration of detailed IC_50_ determination for active compounds is given in Figure 4.

160 kinase inhibitors from a panel of inhibitors known to target kinases in all branches of the kinome were tested for their ability to (i) inhibit LRRK2 at 10 μM in an in vitro kinase assay using the LRRKtide model peptide substrate and (ii) dephosphorylate LRRK2 at phosphoserine 935 in a spotblot assay. Listed are the exact values (expressed as a % relative to the control) obtained in these tests for each compound. A general overview of these tests are given in Figures 1, 3 and the plate and position references given in the first 2 columns correspond to those given in Figures 1B, 3B. Color codes to facilitate identification of compounds from the three selected chemical classes are: (A) 9-methyl-N-phenylpurine-2,8-diamine compounds highlighted in green, (B) N-phenylquinazolin-4-amine analogs given in orange, and (C) 1,3-dihydroindol-2-one analogs in blue. The 20 most potent compounds in the cellular assay are indicated in bold. The 3rd column of the table (labeled “class”) indicates the compound classes (A–C) or the 20 most active compounds in the cellular assay (asterisk), as applicable.

**Table 2:** **Overview of *in silico, in vitro* and cellular activities of selected kinase inhibitors**.

**Compound**	**Compound class**	**GBVI/WSA dG Score**	**pIC50 *in vitro* (lrrktide)**	**IC50 *in vitro* (lrrktide, nM)**	**Cellular pS935 (% @ 5 μM)**	**pIC50 cellular (pS935)**	**IC50 cellular (pS935, nM)**
AG 1478	B	0.6074	5.43	3732.50	75.11	NA	NA
Aminopurvalanol A	A	−5.7089	5.69	2027.68	121.05	NA	NA
Aurora kinase inhibitor II	A	0.0905	<5	>1E4	69.85	NA	NA
Bohemine	A	−4.9590	<5	>1E4	83.36	NA	NA
BPIQ-I	B	−3.5711	5.12	7649.22	88.55	NA	NA
Cdk1 inhibitor	C	−5.6508	<5	>1E4	78.35	NA	NA
Cdk1 inhibitor. CGP74514A	A	−5.4352	5.46	3467.37	83.35	NA	NA
CDK2 inhibitor III	A	−5.9534	<5	>1E4	100.49	NA	NA
Compound 52	A	−4.9190	5.41	3926.45	76.44	NA	NA
Compound 56	B	−2.8627	5.52	3013.01	86.56	NA	NA
DMBI	C	−5.5592	5.83	1482.81	54.34	NA	NA
EGFR/ErbB-2 inhibitor	B	0.3668	<5	>1E4	67.43	NA	NA
GSK-3 inhibitor IX	C	−3.9610	5.95	1122.02	104.53	NA	NA
GSK-3 inhibitor X	C	−4.3149	<5	>1E4	106.06	NA	NA
IC261	C	−4.7642	<5	>1E4	78.81	NA	NA
Indirubin derivative E804	C	−6.0607	6.16	685.49	101.13	NA	NA
Indirubin-3'-monoxime	C	−5.0838	5.75	1786.49	85.53	NA	NA
JAK3 inhibitor II	B	0.2193	5.92	1202.26	63.02	NA	NA
JAK3 inhibitor VI	C	−5.6557	6.76	173.78	40.36	<5.7	>2000
Met kinase inhibitor	C	−4.7526	7.37	42.95	52.43	NA	NA
NF-KB activation inhibitor	B	0.7201	<5	>1E4	65.67	NA	NA
PD 174265	B	−3.4813	5.05	8871.56	67.74	NA	NA
PDGF receptor tyrosine kinase inhibitor III	B	−1.7337	<5	>1E4	72.83	NA	NA
PKR inhibitor	C	−5.8928	6.44	363.08	29	5.88	1312.00
PKR inhibitor. negative control	C	−5.9853	6.53	297.17	72.69	NA	NA
Purvalanol A	A	−5.8267	5.34	4570.88	121.26	NA	NA
Src kinase inhibitor I	B	1.8876	6.03	936.22	61.51	NA	NA
SU11652	C	−7.5028	7.96	10.89	14.19	6.50	316.23
SU6656	C	−7.3049	8.00	10.00	14.34	6.37	422.67
SU9516	C	−5.8674	5.68	2103.78	94.62	NA	NA
Syk inhibitor	C	−6.5548	6.20	632.41	57.29	NA	NA
VEGF receptor 2 kinase inhibitor I	C	−6.6034	7.61	24.55	60.83	NA	NA
VEGF receptor 2 kinase inhibitor II	C	−6.4887	7.44	36.31	37.26	<5.7	>2000
VEGF receptor 2 kinase inhibitor III	C	−5.4369	6.56	272.90	45.08	<5.7	>2000
VEGF receptor tyrosine kinase inhibitor III. KRN63	B	−0.9464	<5	>1E4	62.48	NA	NA
Cdk1/2 inhibitor III	NA	NA	9.04	0.91	13.15	8.93	1.18
Staurosporine	NA	NA	9.57	0.27	8.89	8.76	1.75
LRRK2 IN1	NA	NA	8.45	3.54	9.32	6.55	279.90
CZC25146	NA	NA	8.75	1.78	8.62	7.36	43.65

Compounds belong to one of three different structural classes (A, 9-methyl-N-phenylpurine-2,8-diamine, B, N-phenylquinazolin-4-amine, or C, 1,3-dihydroindol-2-one analogs, see second column) as well as selected reference compounds. In silico docking values given are GBVI/WSA dG. In vitro activities and cellular activities as measured by the LRRKtide assay and phosphoserine 935 assay, respectively, are given both as pIC50 as well as the corresponding IC50 expressed in nM. In vitro inhibition values at the single dose of 10 μM and cellular phosphorylation levels at 5 μM are given in Table 1 for all 160 compounds of the kinase inhibitor panel, cellular phosphorylation levels at 5 μM are also given here for all compounds in the table. See Results and Materials and Methods sections for more details. NA, not applicable.

### Testing of kinase inhibitor panel in LRRK2 cellular phosphorylation assay

**Figure 4:**
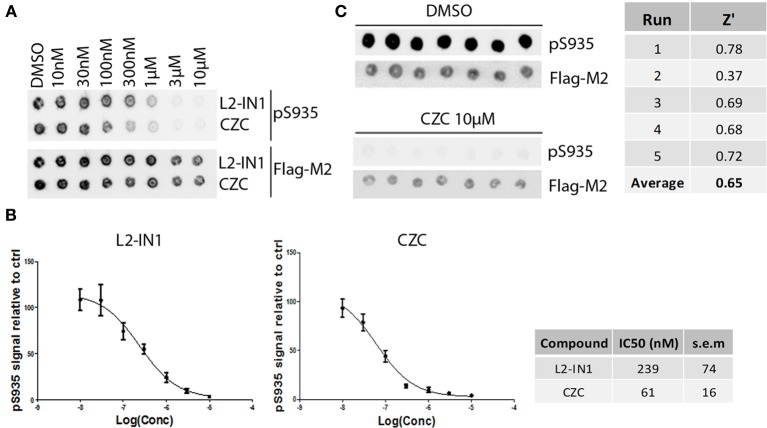
**Dose-response curves and Z' factor determination for Ser935 cellular dephosphorylation assay. (A)** Representative spotblot of cell lysates of SH-SY5Y stably overexpressing 3flag-LRRK2 WT and treated with a dose range of reference compounds LRRK2-IN1 or CZC using concentrations as indicated. DMSO treatment was included as a control. Total LRRK2 protein was detected with Flag-M2 antibody, phosphorylation at Ser935 with a monoclonal LRRK2 PS935 antibody. **(B)** Fitting of inhibition curves based on spot intensity in **(A)** (determined by densitometric analysis and used to quantify phosphorylation levels relative to total LRRK2 levels) and IC50 determination of phosphorylation at LRRK2 Ser935 for reference compounds LRRK2-IN1 and CZC. *N* ≥ 5. Cellular pS935 dephosphorylation IC50 values for each tested compound are given in Table 2. **(C)** Representative spotblot of cell lysates of SH-SY5Y stably overexpressing 3flag-LRRK2 WT and treated with DMSO or 10 μM CZC. Phosphorylation level values derived from total LRRK2 and phospho-LRRK2 levels quantified via spotblot were used to calculate the Z' of the pS935 dephosphorylation assay using the formula given in Materials and Methods.

Using the same panel of 160 kinase inhibitors, cellular activity was assayed by monitoring dephosphorylation of LRRK2 at Ser935 induced by kinase inhibitor treatment (5 μM for 2 h) of the SH-SY5Y cell line with stable expression of LRRK2 as described in Materials and Methods and shown in Figure 3. The mean Z' factor for the dual detection immune-dotblot assay used here is 0.65 (Figure 4). A total of 20 compounds were found to reduce Ser935 phosphorylation levels to less than 50% of control levels (Tables 1, 2), all are ATP-binding site competitive compounds. None of the 20 non ATP-competitive compounds of the panel (see Materials and Methods) induce more than 50% dephosphorylation of LRRK2 at 5 μM although AG490 shows 49.32% dephosphorylation of LRRK2 at 5 μM. Representative dot blot images and bar diagrams are depicted in Figure 3, exact quantifications are given in Table 1. For the selected compounds, the IC50 was determined (Figure 4 and Table 2).

### Correlation between *in vitro* and cellular activity of compounds

**Figure 5:**
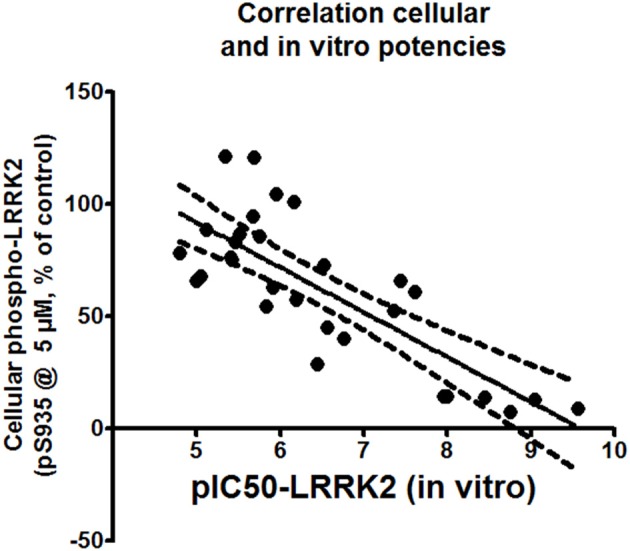
**Correlation plot of cellular activity (pS935 spotblot assay) and *in vitro* potency (pIC50 of LRRKtide assay).** The assessment is performed for compounds with *in vitro* pIC50 of 5 or better. Exact values of the plotted data are given in Table 2.

Correlations between the *in vitro* and cellular activities for each compound were investigated by drawing up correlation plots for these two parameters and performing linear regression analysis as described in Materials and Methods (Figure 5). This analysis showed a significant correlation between *in vitro* and cellular activity (with Pearson's r coefficient of −0.7953).

### LRRK2 kinase structural model

**Figure 6:**
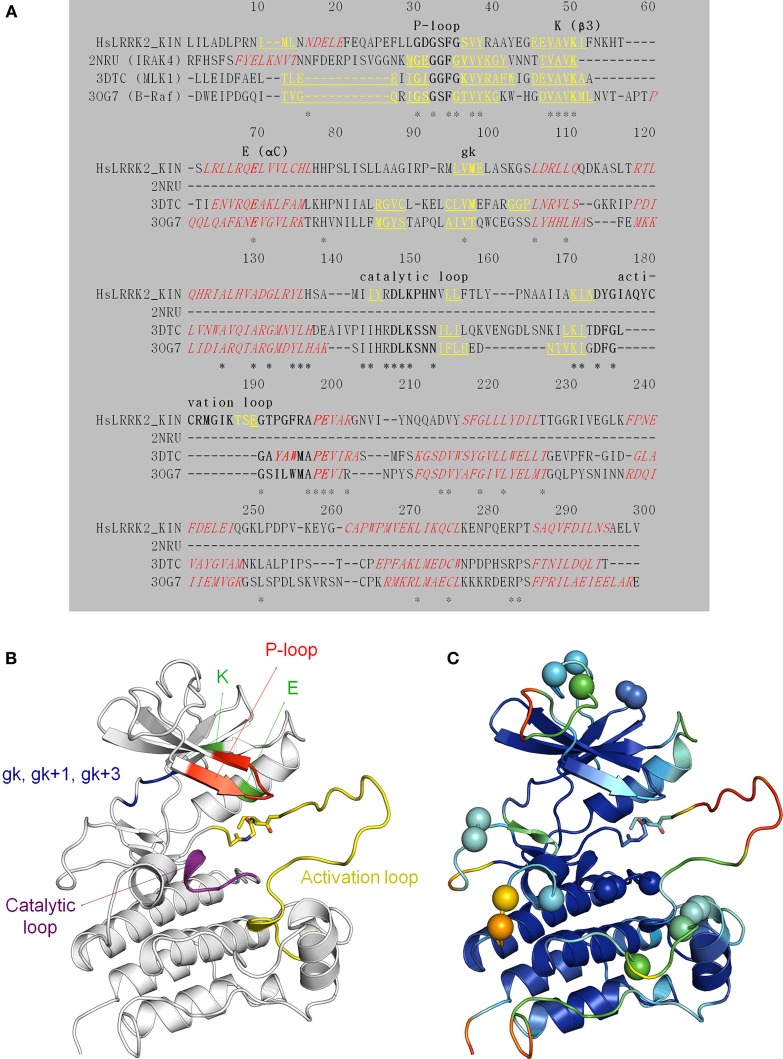
**Structural modeling of the LRRK2 kinase domain (residues 1859–2138). (A)** Target (the LRRK2 kinase domain)—template (2NRU, 3DTC, and 3OG7) alignment (see Table 3 for an overview of available structural templates of TKL kinases). Secondary structure elements (predicted for LRRK2 via NetSurfP or obtained from the 3D PDB structure) are indicated: α-helices are red *italic*, and β-strands yellow underlined. The conserved motifs are highlighted in **bold** and labeled. Identical residues are marked with an asterisk (^*^). **(B)** Illustration of the LRRK2 kinase homology model depicting key residues and functional features. The P-loop is shown in red, the conserved K in β 3 and E in αC in green, the catalytic loop in purple, the activation loop in yellow, the gatekeeper (gk), gk + 1, and gk +3 residues from the hinge region in blue. Sticks colored by CPK convention correspond to the residues that are affected by the G2019S and I2020T mutations segregating with PD. G2019S is part of the beginning of the activation loop. **(C)** Graphical representation of potential errors in the homology model as predicted by Meta-MQAPII. The color spectrum from blue to red reflects the accuracy of the 3D residue prediction from correct to incorrect respectively. The spheres indicate the position of the residues in the disallowed and generously allowed regions of the Ramachandran Plot and residues with unfavorable bond angles and dihedrals. Figures generated with PyMol.

We constructed, optimized and quality improved a 3D homology model of the LRRK2 kinase domain as described in detail in Materials and Methods. Based on their sequence identity with the LRKK2 kinase domain, the tyrosine-kinase like (TKL) kinases B-Raf (PDB 3OG7; Bollag et al., 2010), MLK1 (PDB 3DTC; Hudkins et al., 2008), and IRAK-4 (PDB 2NRU; Wang et al., 2006) were selected as templates to model LRRK2 kinase (see Table 3 for an overview of TKL kinases with available 3D structures and their sequence identity with the LRRK2 kinase domain). The alignment between the LRRK2 kinase domain and these three kinases is given in Figure 6A. The final homology model colored by conserved kinase motifs is shown in Figure 6B; the final model colored by quality of each predicted amino-acid position is given in Figure 6C and was determined as described in the Materials and Methods section. The ATP-binding groove lies at the interface of the N-and C-terminal lobes (Huse and Kuriyan, 2002; Nolen et al., 2004).

**Table 3:**
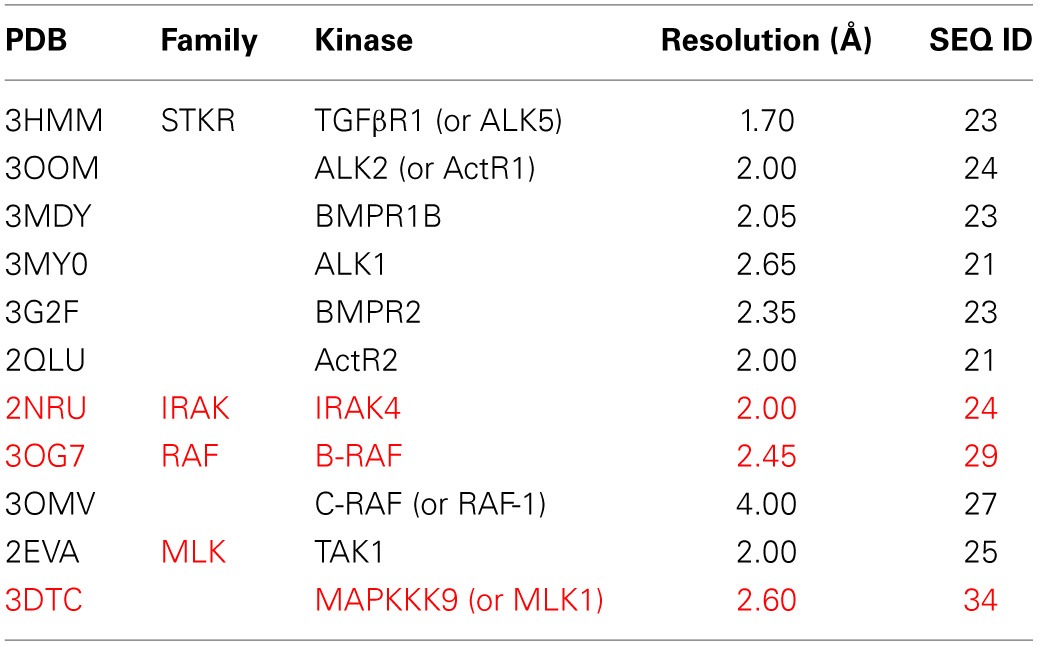
**Overview of TKL kinases with a DFG in conformation available in the MOE “Kinase Database” and their sequence identity with the LRRK2 kinase domain**.

### *In silico* analysis of LRRK2 kinase—ligand interactions

**Figure 7:**
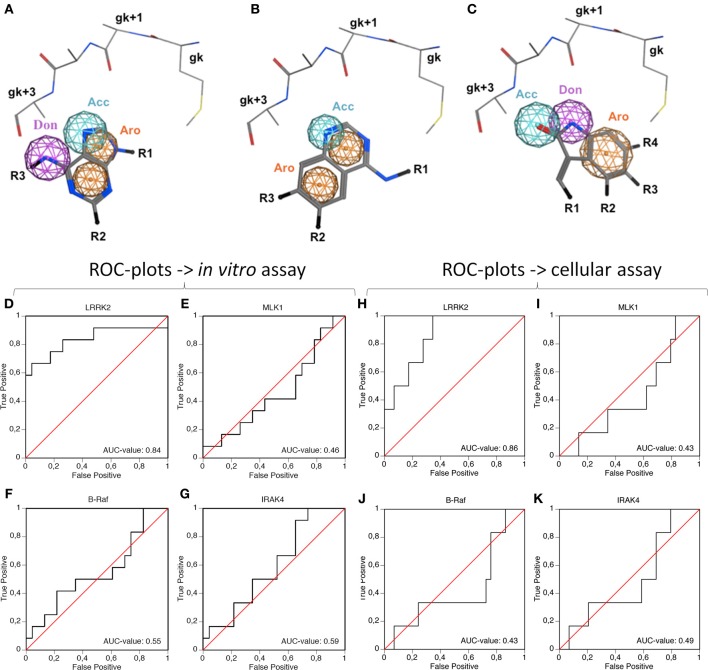
**Illustration of the pharmacophore queries used during “pharmacophore filtering” and receiver operating characteristic plots assessing the correlation between inhibitor docking and *in vitro* or cellular activity. (A–C)** Pharmacophore queries are illustrated for purine, quinazoline and oxindol derivatives [termed compound classes **(A–C)**, respectively, panels **(A–C)**, respectively]. Shown are the hinge region residues, pharmacophore features and the substructure of the respective class. Only the main chain and Cβ side chain atoms are shown for the gk-1, gk+1, gk+2, gk+3, and gk+4 residues. Depicted pharmacophore features include aromatic rings (orange spheres), hydrogen bond acceptors (cyan spheres), or donors (purple spheres). The substructures of the respective compound classes are shown in stick representation. All molecules are colored by CPK convention. Abbreviations: Acc, acceptor; Aro, aromatic; Don, donor; gk, gatekeeper. Figures generated with MOE. **(D–K)**. The receiver operating characteristic plots were determined to evaluate the predictive power of the docking method in the LRRK2 model compared to the separate templates used to generate the LRRK2 model, i.e., B-Raf (3OG7), MLK1 (3DTC), and IRAK-4 (2NRU). Using the activity measures for *in vitro* activity **(D–G)** or cellular activity **(H–K)**, true positive rate (measure for sensitivity) as function of false positive rate (indication of specificity) is plotted. The Area Under Curve (AUC)-value of each receiver operating characteristic plot is indicated on the plot, with an AUC > 0.5 indicating predictive value of the docking. **(D,H)** receiver operating characteristic plots of LRRK2 docking, **(E,I)** receiver operating characteristic plots of MLK1 docking, **(F,J)** receiver operating characteristic plots of B-Raf docking, **(G,K)** receiver operating characteristic plot of IRAK4 docking. Docking scores are given in Table 2.

**Figure 8:**
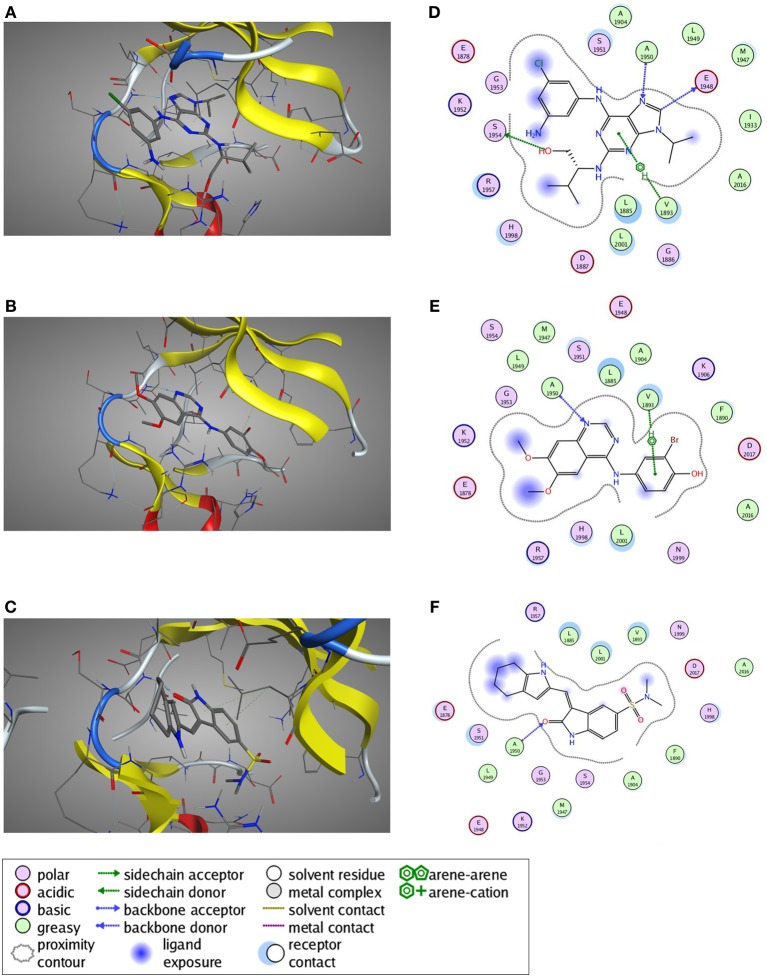
**Predicted ligand binding poses. (A–C)** Selected active compounds of the three different compound classes are depicted in the LRRK2 kinase ATP-binding pocket. Compounds shown are **(A)** Aminopurvalanol A (purine derivative), **(B)** JAK3 Inhibitor II (quinazoline derivative), and **(C)** SU6656 (oxindole derivative). LRRK2 kinase is given in cartoon and line representation, the ligands in stick representation. The blue dotted lines denote hydrogen bond interactions. The green dotted lines denote π —cation interactions. **(D–F)** Schematic representation of the interactions between the LRRK2 kinase active site and the compound from panels **(A–C)**: **(D)** Aminopurvalanol A, **(E)** JAK3 Inhibitor II, and **(F)** SU6656. See box for the explanation of colors and arrows. Figures generated with MOE.

A preliminary docking step, where staurosporine was docked in the LRRK2 ATP-binding site, was applied to optimize the local environment to get the most optimal binding pose during the subsequent docking step. 35 compounds from three compound classes (A) 9-methyl-N-phenylpurine-2,8-diamine, (B) N-phenylquinazolin-4-amine, and (C) 1,3-dihydroindol-2-one analogs were docked into this active site using three class-specific pharmacophore models (Figures 7A–C) as described in detail in Materials and Methods. A summary of the *in silico* docking scores, given as the Generalized-Born Volume Integral/Weighted Surface area dG (GBVI/WSA dG) is given in Table 2. To evaluate the correlation between docking and *in vitro* and cellular compound activities, receiver operating characteristic plots describing the trade-off between sensitivity and specificity were constructed using the GVBI/WSA dG computed values and the measured *in vitro* activities in the LRRKtide assay (IC50 of 1 μM or better is scored active) or the measured pS935 cellular dephosphorylation (a measure of greater than 50% dephosphorylation at 5 μM is scored as active). Receiver operating characteristic plots which trace above the diagonal signify docking enrichment, with best docking for those receiver operating characteristic plots furthest above the diagonal. An indicative parameter of the receiver operating characteristic plot is the area under the curve (AUC) with values above 0.5 indicating a valid correlation between *in silico* and measured activity values. The receiver operating characteristic plots were determined for the LRRK2 kinase model as well as the three separate kinase structures which were used as templates for constructing the LRRK2 kinase model. These receiver operating characteristic plots, displayed in Figures 7D–K clearly show that of these four models, only docking results obtained with the LRRK2 kinase domain model itself have predictive value for *in vitro* kinase activity and cellular pS935 dephosphorylation activity for at least three different structural classes of LRRK2-active compounds. Illustrative LRRK2 kinase—ligand binding poses for representative active compounds representing the three different classes are given in Figure 8.

## Discussion

The relationships between binding of kinase inhibitors to the LRRK2 kinase pocket, *in vitro* inhibition of LRRK2 kinase activity and inhibitor-induced cellular dephosphorylation of LRRK2 hold clues for understanding LRRK2 signaling and interpreting LRRK2 cellular activity assays. Here, we assessed the interrelationship between these parameters, using *in silico, in vitro* and cellular activity assays. Using a panel of 160 reference kinase inhibitors targeting all branches of the kinome, we found a broad range of potencies to inhibit LRRK2 *in vitro* kinase activity, ranging from inactive compounds to compounds inhibiting LRRK2 in the subnanomolar range. Similarly, the panel of kinase inhibitors displayed a broad range of cellular potencies with strongest compounds active in the low nanomolar range. Finally, the picture of activities was completed by determining *in silico* docking of compounds selected from three different structural classes which included compounds with identical scaffolds but varying activities ranging from potent to inactive in the *in vitro* kinase assay and cellular dephosphorylation assay.

The activity assays selected and optimized here are suitable to predict activity of compounds on LRRK2. First, for the *in vitro* activity and cellular activity assays, we used established assays with slight modifications, and we could demonstrate excellent Z'-factors for these assays in our hands (Z' of 0.82 for the *in vitro* assay and of 0.65 for the cellular assay). The results obtained with LRRKtide *in vitro* phosphorylation assay as performed here yielded potencies similar to those reported for compounds tested in previously published studies. Using LRRK2-IN1 as a benchmark, the IC50 value obtained here of 3.54 nM is comparable to the published value (13 nM) (Deng et al., 2011). The same holds true for the second assay testing cellular activity which is based on kinase inhibitor induced dephosphorylation of LRRK2 at Ser935 and adapted here using a spotblot readout. For instance, for LRRK2-IN1 the IC50 value obtained here (279.9 nM) is in the same range as IC50 values obtained with other readouts of pS935 levels such as western blot (about 100 nM; Dzamko et al., 2010; Deng et al., 2011), time-resolved FRET (90–200 nM; Hermanson et al., 2012), or ELISA (50–100 nM; Delbroek et al., 2013). Thirdly, in the absence of a physical 3D atomic structure of the LRRK2 kinase domain, an optimized 3D homology model was generated for *in silico* docking. Because the available templates with closest homology showed only 30% sequence identity, we took great care in optimizing the model. Homology models of the LRRK2 kinase domain have been constructed before (see for example references; Marin, 2006; Mata et al., 2006; Nichols et al., 2009; Deng et al., 2011; Yun et al., 2011), however here we refined the model extensively for use in molecular docking studies. In particular, alignments between LRRK2 kinase and potential templates were performed using alternative sequence-to-structure alignments guided by conserved residues Also, multiple templates were used to employ the most appropriate template for each structural segment. Finally, the best model was chosen according to the model evaluation rather than the alignment score.

Our results show a strong correlation between the potency of compounds to inhibit LRRK2 *in vitro* kinase activity and potency to dephosphorylate LRRK2 in cells (Pearson's *r* = −0.7953, Figure 5). Kinase inhibitor induced dephosphorylation of LRRK2 in cells could be seen for compounds with an *in vitro* IC50 of 10 μM or better. Conversely, the majority of the compounds which were inactive *in vitro* (no activity at 10 μM) were also inactive in cells. This is consistent with the notion that kinase inhibitors induce dephosphorylation through inhibition of LRRK2 kinase activity and/or through binding to the LRRK2 kinase ATP binding pocket. This conclusion is supported by receiver operating characteristic plots generated with the compound docking scores and the *in vitro* potency of each tested compound. It is often observed that in many cases docking based virtual screening performs no better than random selection. Inclusion of pharmacophore methods however has shown to significantly improve the virtual screening performance (Voet et al., 2014). As such we also combine pharmacophore models in our virtual screening setup. Via this approach, docking into our modeled LRRK2 kinase structure is clearly better in discriminating actives from decoys than docking into the three other kinases which were used to generate the LRRK2 model. Therefore, we can conclude that the *in vitro* and cellular activities of the compounds of the three structural classes tested are likely dependent on their binding to the LRRK2 ATP-binding site.

While it is not surprising that LRRK2 kinase activity is inhibited more potently by compounds that bind more tightly to the kinase, this is less evident for the correlation between binding and cellular dephosphorylation of LRRK2. Indeed, kinase inhibition of LRRK2 would be expected to reduce the phosphorylation rate of LRRK2 autophosphorylation sites (Greggio et al., 2009; Gloeckner et al., 2010), however the Ser935 used in the assay is not an autophosphorylation site (Nichols et al., 2010; Doggett et al., 2012; Lobbestael et al., 2012), rather it is a site phosphorylated by other kinases. This has implications for the signaling properties of LRRK2. Indeed, the direct regulation of the phosphorylation/dephosphorylation equilibrium in cells involves two partners, a phosphatase and a kinase. Using the kinase inhibitor signaling panel with broad coverage of the kinome, we expected to identify at least a few compounds which would be inactive on the LRRK2 kinase activity *in vitro*, but active in dephosphorylating LRRK2 as these active compounds would point to the upstream kinases of LRRK2. Looking to Table 1, only a handful of *in vitro* inactive compounds (such as LY303511, DNA-PK inhibitor V, Aurora kinase inhibitor 3, BAY11-7082) could affect moderate dephosphorylation of LRRK2 (~50%). These four compounds are directed against kinases of different branches of the kinome: LY303511 (lipid kinases branch), DNA-PK inhibitor V (atypical kinases branch), Aurora kinase inhibitor 3 (other kinase branch), BAY11-7082 (other kinases branch). Although these compounds were not among the top hits of the study, further characterization of these compounds on their effects in regulating LRRK2 phosphorylation may provide more information on putative upstream kinases of LRRK2. For example, BAY11-7082 is reported to be a specific inhibitor of inducible Iκ B-alpha phosphorylation (Pierce et al., 1997) which is in line with the finding that Iκ B-alpha shown to phosphorylate LRRK2 in immune cells (Dzamko et al., 2012). However, none of the other *in vitro* inactive compounds displayed significant dephosphorylation of LRRK2 in cells. Also, it should be noted that full dephosporylation of LRRK2 (>70–80%) was not displayed by any of the *in vitro* inactive compounds and was only observed for the most potent *in vitro* inhibitors of LRRK2.

Taking the overall results into account, we conclude that LRRK2 dephosphorylation involves an important contribution of the activity of compounds on LRRK2 itself. Therefore, the regulation of LRRK2 phosphorylation at Ser935, as well at other sites of this phosphorylation cluster such as Ser910/955/973, involves at least three partners, i.e., a phosphatase, a phosphorylating kinase as well as LRRK2 itself. We recently showed that treatment of cells with the LRRK2 kinase inhibitor LRRK2-IN1 induced LRRK2 dephosphorylation by recruitment of protein phosphatase 1 (PP1) (Lobbestael et al., 2013). Our findings presented here suggest that this is a more generalized phenomenon for LRRK2 kinase inhibitors from multiple kinase classes. The activity of these compounds may possibly require conformational changes of the LRRK2 kinase domain to allow proper binding of the inhibitor. Experimental evidence for this has recently been reported, whereby detection of LRRK2 with a monoclonal antibody targeting the activation loop of the LRRK2 kinase domain was altered upon binding of kinase inhibitors (Gillardon et al., 2013). A compound induced conformational change of LRRK2 is likely to regulate binding affinities between LRRK2 and its cellular interactors and is consistent with our previous observation that PP1 is recruited to LRRK2 under conditions of dephosphorylation (Lobbestael et al., 2013). It remains to be determined whether compounds can be developed which inhibit LRRK2 kinase activity but which do not induce major conformational changes in LRRK2 leading to its dephosphorylation in cells. This has important implications as the pS935 dephosphorylation is not only observed after LRRK2 kinase inhibition (Deng et al., 2011; Choi et al., 2012; Reith et al., 2012; Zhang et al., 2012), but also in at least some LRRK2 disease mutants (Nichols et al., 2010; Li et al., 2011; Lobbestael et al., 2012; Rudenko et al., 2012), therefore it is not yet clear whether dephosphorylation is a desired effect of a potential PD therapeutic based on LRRK2 kinase inhibition.

In conclusion, we report here the correlations between the *in vitro*, cellular and *in silico* activities of a kinome-wide panel of kinase inhibitors on LRRK2. Our results indicate that cellular LRRK2 dephosphorylation induced by kinase inhibitors involves a strong component of inhibitor activity on LRRK2 itself, without excluding a role for upstream kinases. Understanding the regulation of LRRK2 phosphorylation by kinase inhibitors has implications for cellular activity assays of LRRK2 and may lead to development of new classes of LRRK2 kinase inhibitors.

## Materials and methods

### *In vitro* kinase assay

LRRK2 kinase activity was assessed using an isotopic peptide substrate assay essentially as described in reference (Taymans et al., 2011). In short, recombinant LRRK2 was incubated with 6 μCi γ−^32^P-ATP [3000 Ci/mmol; Perkin Elmer (USA)], 200 μM LRRKtide (RLGRDKYKTLRQIRQ) (Jaleel et al., 2007) [Enzo Life Sciences (USA)], 10 μM ATP and kinase inhibitor (see below) or dimethylsulfoxide (DMSO) solvent per 40 μl reaction in 1× kinase buffer for 30 min at 30°C. The composition of 1x kinase buffer is: Tris 25 mM pH 7.5, MgCl_2_ 10 mM, dithiothreitol (DTT) 2 mM, Triton 0.02%, beta-glycerophosphate 5 mM, Na_3_VO_4_ 0.1 mM. DMSO content of each *in vitro* kinase reaction was 1 %. For the single dose testing (at 10 μM of kinase inhibitor), the LRRK2 enzyme used was GST-tagged truncated LRRK2 containing residues 970 to 2527 (Life Technologies). Reactions were stopped by the addition of 500 mM EDTA containing bromophenol blue. Reactions were spotted to P81 Whatman phosphocellulose paper (GE Healthcare) and washed four times 10 min in 75 mM phosphoric acid. LRRKtide phosphorylation levels were measured via autoradiography (Asensio and Garcia, 2003).

A commercially available panel of 160 kinase inhibitors (EMD4Biosciences, Inhibitor select panel) was initially screened at one concentration (10 μM). The inhibitor panel contains cell-permeable and previously characterized inhibitors which together target all branches of the kinome. All inhibitors are confirmed cell permeable, with the exception of PKCβ Inhibitor, PKR Inhibitor—Negative Control, Alsterpaullone—2-Cyanoethyl, Cdk1/5 Inhibitor and JNK Inhibitor IX which are of unknown permeability. Compounds in the inhibitor panel are mostly ATP-binding site competitive inhibitors, although 20 compounds are labeled as non ATP-competitive compounds (including 1 allosteric compound). These compounds are: Bcr-abl Inhibitor, AG 490, AG 112, Akt Inhibitor X, AG 1024, Akt Inhibitor V—Triciribine, Akt Inhibitor VIII, Isozyme-Selectiv—Akti-1/2, Chelerythrine Chloride, MEK1/2 Inhibitor, MNK1 Inhibitor, KN-62, Cdk4 Inhibitor II—NSC 625987, ERK Inhibitor III, MK2a Inhibitor, MEK Inhibitor I, Sphingosine Kinase Inhibitor, PD 98059, GSK-3b Inhibitor I, KN-93 and the allosteric inhibitor IGF-1R Inhibitor II. Information on the branch of the kinome targeted is provided together with experimental results in Table 1; further details on each inhibitor are available from the supplier. Then, selected molecules were further tested at lower doses. For those selected compounds that were found to inhibit >50% at 1 μM, an IC_50_ (inhibitor concentration yielding 50% inhibition) was determined. To this end, compounds were tested in triplicate with full length LRRK2 protein (Taymans et al., 2011; Civiero et al., 2012) at concentrations of 10 μM, 1 μM, 300 nM, 100 nM, 30 nM, 10 nM, 3 nM, 1 nM, 100 pM, 10 pM, and solvent. An inhibition curve was fitted and IC50s calculated using GraphPad Prism version 5.01 for Windows (GraphPad Software, San Diego, CA, USA). IC50s are expressed as pIC50 [=−log(IC50)].

### LRRK2 cellular activity and phosphoserine 935 spotblot detection assay

To assess LRRK2 cellular activity, an SH-SY5Y stable cell line was first generated. SH-SY5Y cells were cultured in Dulbecco's modified Eagle's medium supplemented with 15% fetal bovine serum and 1× non-essential amino acids (Gibco) at 37°C and 5% CO_2_. Lentiviral vectors (LVs) encoding human 3xFlag-LRRK2 under control of the cytomegalovirus (CMV) promoter and co-expressing a hygromycin selection marker via an internal ribosomal entry site element were prepared and used for cellular transduction as previously described (Civiero et al., 2012). Following selection in medium containing 200 μg/ml hygromycin, cells were expanded for use in experiments.

Cells were plated out into 96-well plates. When wells were >80% confluent, cells were treated with kinase inhibitors by dilution of the compounds into the cell culture medium to the desired final concentration. Following a 2 h incubation of the cells with kinase inhibitors, cells were immediately rinsed in PBS and lysed in lysis buffer [20 mM Tris-HCl pH 7.5, 150 mM NaCl, 1 mM EDTA, 0.5% Tween 20 or 1% Triton X-100, 2.5 mM sodium pyrophosphate, 1mM beta-glycerophosphate, 1 mM NaVO_4_, protease inhibitor cocktail and phosphatase inhibitor cocktail (Roche)]. Lysates were centrifuged for 30 min at 14000 × g. Supernatant was spotted to hydrated pvdf membranes and LRRK2 phospho-Ser935 levels as well as total LRRK2 levels were sequentially determined by immunoblot detection using the rabbit monoclonal anti-phospho-S935-LRRK2 antibody [Epitomics, clone UDD2 10(12)] and the mouse monoclonal anti-LRRK2 antibody N138/6 (Neuromab) or flag-M2 antibody followed by incubation with appropriate secondary horseradish peroxidase coupled antibodies and chemiluminescent detection using ECL plus HRP substrate (Thermo Scientific, Rockford, IL, USA). Densitometric analysis of the immunoreactive spots was performed using Aida analyzer v1.0 (Raytest, Straubenhardt, Germany). Phosphorylation levels were determined by the ratio of phospho-LRRK2 to total LRRK2, normalized to solvent controls.

### Z' determination

To determine Z' of the *in vitro* LRRKtide assay or the cellular Ser935 dephosphorylation assay, the following formula was used:

$$Z\prime =1-\left(\frac{3\sigma_{+c}+3\sigma_{-c}}{\left|\mu_{-c}-\mu_{+c}\right|}\right)$$

where σ_+c_, σ_−c_, μ_+c_, and μ_−c_ are the standard deviation (σ) and mean (μ) of the positive control samples (+c, LRRK2-IN1 10 μM for the LRRKtide assay, CZC 10 μM treated samples in the pS935 dephosphorylation assay) or negative control samples (−c, DMSO treated samples). Results are based on values from 3–10 replicates from the same assay run.

### Modelling the LRRK2 kinase domain based on multiple templates

All the following steps were conducted using MODELLER 9v9 (Sali and Blundell, 1993), unless otherwise stated. First, human TKL kinases with a DFG-in activation loop conformation (i.e., active conformation) available at that time in the “Kinase Database” implemented in Molecular Operating Environment (MOE, Chemical Computing Group Inc., 1010 Sherbooke St. West, Suite #910, Montreal, QC, Canada, H3A 2R7, 2013) were structurally aligned using the alignment.salign command. Afterwards, this structure domain was superimposed on the LRRK2 kinase domain. The TKL kinases B-Raf (Rapidly accelerated fibrosarcoma) (PDB 3OG7; Bollag et al., 2010) and MLK1 (Mixed Lineage Kinase 1) (PDB 3DTC; Hudkins et al., 2008) and—for one short stretch of 11 amino-acids in the N-terminal region of LRRK2 kinase (corresponding to LRRK2 amino acids 1872–1882 found just N-terminal of the P-loop, see Figure 6A)—IRAK-4 (Interleukin-1 Receptor–Associated Kinase 4) (PDB 2NRU; Wang et al., 2006) displayed the highest identity with the LRRK2 kinase domain and were chosen as templates. The final alignment between B-Raf, MLK1, IRAK-4, and the LRRK2 kinase domain is shown in Figure 6A. A 3D model of LRRK2 kinase domain was calculated by satisfaction of spatial restraints and screened for unfavorable regions by computing the Discrete Optimized Protein Energy (DOPE) score per residue. The alignment was modified using iterative alignment-modeling-evaluation steps until no improvement could be found. For the top-scoring alignment, multiple models were computed and subjected to a rough refining procedure: each model is optimized with the variable target function method with conjugate gradients and further refined using molecular dynamics with simulated annealing. The LRRK2 kinase domain model with the best DOPE score, GA341 score and Modeler objective function was selected.

Explicit hydrogen atoms were added and the model was subjected to a more thorough refining procedure with MOE using the AMBER99 force field with Born solvation model. First, all inconsistencies and outliers were selected (as observed from values in the Ramachandran plot, backbone bond angles and lengths, the rotamer strain energy, and atom clashes), the other residues were potentially fixed and an energy minimization (EM) was performed with backbone atoms restrained to 100. The EM was terminated when the RMS gradient fell below 0.1. A final EM was performed on all atoms with all backbone atoms restrained to 10. This minimization was terminated when the RMS gradient fell below 0.1.

### Quality

Model quality has been checked by computational methods, giving us a good validation of the reliability of the model. The Ramachandran Plot assured very good confidence: only 0.7% residues in the disallowed region and 2.1% residues outside generously allowed regions. 2.9% of the residues had unfavorable bond angles and 1.1% had unfavorable dihedrals. However, most of these residues were oriented away from the active site (Figure 6C). Assessment of model quality using Meta-MQAPII (Pawlowski et al., 2008) gave absolute global deviations, expressed as RMS deviation (3.47 Å) and Global Distance Test Total Score (65.98), for the model vs. the unknown true structure, indicating a medium quality model. The Meta-MQAPII score per residue is shown in Figure 6C. The only unfavorable regions were the loop regions, especially the activation loop, explained by the lack of a good template for these regions (Figure 6A). Overall, the activation loop is also rather flexible for kinases (un-crystallized region). Since these unfavorable modeled loops are not part of the ligand binding site, we proceeded with the LRRK2 kinase domain homology model. PyMOL and MOE were used as a visualization tool. The model is freely available upon request to the corresponding authors.

### Preparation of the *in silico* kinase inhibitor database

The kinase inhibitor database was supplied as a two-dimensional structure data file by EMD4Biosciences (USA). Using the MOE Structure Database Tools (sdwash, sdcharges, and sdstereo commands), the database was curated. Based on the fact that different stereoisomers may have different activities, molecular docking simulations were carried out for both stereoisomers. Generation of 3D structures was done via the energy minimize command using default settings. We used the MMFF94x force field. All data were stored in a MOE molecular database file.

Common substructures, based on the analysis of PDB kinase ligands by Ghose and co-workers (Ghose et al., 2008), were found using Instant JChem (ChemAxon, Hungary).

### Protein-ligand docking and scoring

After homology modeling with MODELLER (Sali and Blundell, 1993), the generated alternative conformations of the ligands were docked into the active site using MOE. A preliminary docking step, where staurosporine was docked in the LRRK2 active site, was applied to optimize the local environment to acquire the most optimal binding pose in the subsequent docking step. The crystal structures B-Raf (PDB 3OG7), MLK1 (PDB 3DTC), and IRAK-4 (PDB 2NRU) were protonated using Protonate3D module of MOE.

For docking simulations, initial binding conformations were generated for the purine, quinazoline, and oxindol derivatives (termed compound classes A–C) of the kinase inhibitor database. These initial binding conformations were refined using pharmacophore models for these three compound classes (Figures 7A–C). The pharmacophore models were based on structural elements, shared by all derivatives in one class that are essential for interaction with LRRK2. During the refinement step, ligands that fulfilled the pharmacophore hypothesis were allowed to advance and an optimized binding conformation was saved in a MOE molecular database file. Ligands that didn't satisfy the pharmacophore requirements were excluded from subsequent steps. The DOCK module was used and default settings were applied. A force field based scoring function was used: GBVI/WSA dG. After docking, the results were collected by receptor (i.e., docking values obtained with each of the four kinase structures tested). For each ligand the best scoring (e.g., lowest energy) docking pose was kept.

### Receiver operating characteristics plots

Receiver operating characteristic plots are useful as a graphical illustration of the performance of the *in silico* docking strategy as they can evaluate the computed docking values together with measured activities (either *in vitro* or cellular). Here, we plotted the receiver operating characteristic plots as false positive rate (equivalent to the 1—*specificity)* vs. the true positive rate (equivalent to the sensitivity), therefore docking strategies which plotted on average above the diagonal can be considered to have predictive value (this is also reflected by the AUC which is >0.5 for predictive docking strategies). In more detail, for each ligand, the activity/inactivity was indicated by adding 1 and 0 respectively based on the *in vitro* LRRKtide assay or cellular pS935 dephosphorylation assay. Ligands with at least 50% inhibitory activity at 1 μM in the LRRKtide assay or at least 50% inhibitory activity at 5 μM in the cellular pS935 dephosphorylation assay were designated as active. A receiver operating characteristic plot was generated for each receptor (i.e., for each kinase model) using CROC v1.0 (Swamidass et al., 2010). Plots were made with Deltagraph V7 (2014 RedRock Software, Salt Lake City, USA).

### Correlation analysis

To evaluate the correlation between the different activities obtained for tested kinase inhibitors, pairs of activity values were plotted against each other in GraphPad Prism 5.01 (GraphPad Software Inc.). Linear regression analysis was performed and a trendline was drawn as well as the 95% confidence band. Finally, Pearson's r-coefficient was calculated and a two tailed correlation significance test performed (GraphPad). The level of statistical significance was set at *P* < 0.05.

### Conflict of interest statement

The authors declare that the research was conducted in the absence of any commercial or financial relationships that could be construed as a potential conflict of interest.
